# Some of the Properties of Hydrogen Peroxide Solutions

**Published:** 1893-09

**Authors:** Henry P. Talbot


### SOME OF THE PROPERTIES OF HYDROGEN PEROXIDE SOLUTIONS.[1]

BY HENRY P. TALBOT, PH.D.[2]

THERE has been in recent years a marked decrease in the use of the solution of hydrogen peroxide as a detergent and aid in the removal of diseased tissue, and the peroxide itself has fallen into disrepute as a result of the failures attending the use of commercial solutions by dental practitioners. But it is an open question whether this fate is not rather deserved by those who have claimed to furnish the market with a reliable article than by the peroxide itself.

At the suggestion of some of the members of your Society I have undertaken to appear in defence of the peroxide,—at least so

[1] Read before the Harvard Odontological Society, April 27, 1893.

[2] Massachusetts Institute of Technology.

far as chemical evidence is concerned,—to make some statements regarding the nature of our commercial solutions, and to show what the chemical properties of properly-prepared solutions of this useful reagent really are. The subject will be treated from a chemical stand-point chiefly.

Hydrogen peroxide is represented chemically by the symbol $H_2O_2$; that is, its molecule is composed of two atoms each of hydrogen and oxygen. The similarity of this molecule to that of water, $H_2O$, is at once apparent, and has led to the application of the name "oxygenated water" to the compound, a title which is misleading, as is indicated by the publication of erroneous statements based upon the assumption that the solutions of hydrogen peroxide are merely water charged with oxygen or ozone. Such is not the case. The hydrogen peroxide exists in solution in the water in the same way as common salt in a brine.

The best of our knowledge at present leads us to assume that within the molecule of the peroxide the two oxygen atoms are joined together, and that each has a hydrogen atom attached to it, thus, H—O—O—H. This intermolecular arrangement is one which frequently results in the production of unstable compounds, to which class the hydrogen peroxide belongs. Indeed, it is this very lack of stability which constitutes the valuable property of the peroxide, inasmuch as it leads to a ready decomposition into water and oxygen; $2H_2O_2 = 2H_2O + O_2$. The higher oxide ($H_2O_2$) breaks up into the lower oxide ($H_2O$) and oxygen gas ($O_2$), the latter being liberated. This oxygen is, at the moment of its liberation, nascent oxygen, or oxygen in its state of greatest efficiency. In this connection a word or two regarding our conceptions of the difference between nascent oxygen and gaseous oxygen may be of interest. Gaseous oxygen is made up of great numbers of oxygen molecules, and these molecules in turn are made up of oxygen atoms. We have reason to believe that there are two oxygen atoms in each molecule. Before a molecule of oxygen can become chemically active, the parts must be torn asunder; that is, the atoms must first be separated before they react. This requires a certain amount of energy, and the energy thus consumed lessens that of the chemical action. But at the moment of liberation from the hydrogen peroxide the oxygen is in its atomic or nascent state. The liberated atom has not yet entered into combination with any other atom to form a molecule; it is in its nascent condition, and at that instant it is ready to display its greatest chemical activity, since no energy is to be expended in tearing apart the molecule as indicated above.

Fortunately, simple contact with diseased or dead tissue is sufficient to cause decomposition of the hydrogen peroxide, and the nascent oxygen liberated at once attacks and destroys the waste material. The process is identical in its nature with that of combustion.

The peroxide works in a friendly spirit, for while the waste material is promptly attacked, the healthy tissue is acted upon slightly, or not at all. This may be easily seen by placing a drop of the solution upon the skin and observing the evolution of oxygen resulting from the action of the peroxide upon the waste material which is always present.

The intensity of the action depends upon the concentration of the solutions employed, and if the more concentrated solutions to which reference will be made are used, the results are twofold. In the first place, the diseased tissue is attacked, and the mechanical removal either completely accomplished or at least facilitated, while, in the second place, the solution acts as a germicide. The germicidal action takes place, too, under the most favorable conditions,—namely, not merely on the surface, but within the coating, a point of much importance. Reference will be made to this again.

Among the properties of hydrogen peroxide which make it of special value to the dental and medical profession should be mentioned its non-poisonous character and the harmless nature of its decomposition products, oxygen and water. In this respect it is distinctly preferable to corrosive sublimate, carbolic acid, and the like.

Unfortunately, the hydrogen peroxide does not always wait for the presence of oxidizable substances before undergoing decomposition. Spontaneous decomposition takes place slowly, to which is due, in a large measure, the poor quality of the solutions to be found in the market. But there is a generally mistaken notion abroad that this deterioration is so rapid as to render the preservation of solutions practically an impossibility. This is not so. A little care, and a knowledge of the conditions which tend to cause decomposition, will enable dealers to keep a small stock on hand in fair condition for some time. These solutions cannot, however, be treated as the majority of other preparations are treated, and must not be kept for a long period in stock, particulary during the summer months. Undoubtedly freshly-prepared solutions would be kept on hand and sold if a demand were always made for strictly first-class preparations. There is, unfortunately, no easy and reliable chemical method which dentists can use to test the value of solutions offered. They are obliged to judge from their practical efficiency.

The commercial solutions of the peroxide should range from eighteen to twenty-three volumes. In point of fact, I have found them to vary from four to twenty-three volumes. On four occasions I have found solutions, sold in this city for eighteen-volume solutions, which were actually less than ten volumes, two of them four volumes each. Inasmuch as such solutions are little, if any, better than an equal quantity of water, it is not strange that dentists, into whose hands such solutions may come fail to obtain satisfaction from their use, and denounce the remedy as a whole.

Other remedies are subject to the same variations in value as the hydrogen peroxide. Some of these have come under my notice, and the results of my examination of several medicinal preparations lead me to suggest that it is not safe to depend upon the quality of the reagents purchased in the market to-day, and failure to attain expected or predicted results is not always to be attributed to misstatements on the part of the founder of a new method of treatment, but the cause is often to be sought in the quality of the article offered for sale by the druggist or other dealer in supplies.

There are two systems in common use to express the value of hydrogen peroxide solutions. The American custom designates as a twenty-volume solution one which, when brought into contact with a substance which will give up an atom of oxygen to unite with each atom furnished by the peroxide, will evolve twenty times its own volume of oxygen gas. Potassium permanganate is a representative of the class of substances referred to, and one cubic centimetre of a peroxide solution, when poured into an acid solution of the permanganate, should evolve twenty cubic centimetres of oxygen to accord with the American definition of a twenty-volume solution. The English custom requires that one cubic centimetre of a solution shall, of itself alone, by decomposition, yield twenty cubic centimetres of oxygen in order to be designated as a twenty-volume solution. It is obvious that the second solution is twice as strong as the first. As Dr. F. H. Williams points out in a recent number of the *Boston Medical and Surgical Journal*,[1] much confusion would be avoided by the use of percentages to express the amount of peroxide present in the solutions. A twenty-volume solution (American system) corresponds to about three per cent. by weight; a fifty-volume solution to 6.9 per cent.[2]

---

[1] Vol. cxxvii. p. 303.

[2] This system has recently been adopted by the manufacturers of the so-called "pyrozone" solutions.

It has already been stated that the energy of the action of the peroxide is greater the greater the concentration. It is of advantage to be able to accomplish much with a small quantity of the solution, and for such cases solutions of greater strength than the commercial solutions may be employed with success. Moreover, it appears that the germicidal action of the peroxide is not vigorous until the concentration has reached about fifty volumes. The only data available regarding the germicidal power of hydrogen peroxide are those published by Dr. Williams in the paper already referred to, where he describes the application in cases of diphtheria, in which the part played by the peroxide is, in a general way, similar to its rôle in dentistry. From the experiments quoted it appears that a strength of fifty volumes is required before the germicidal action is prompt and complete.[1]

It is perfectly possible to prepare solutions which will evolve three hundred volumes, or over, of oxygen, and fifty-volume solutions may be kept in stock for short periods without appreciable deterioration. The cost of such solutions is necessarily greater in proportion than the commercial preparations, owing to loss incidental to their manufacture, and the difficulty in maintaining them at their proper strength. The increased cost would be, I imagine, a minor consideration, if the remedy proved to be satisfactory in itself.

The concentration is accomplished by distillation at reduced pressure, and the maximum strength reached in my experiments was five hundred and eighty volumes. If the pressure is reduced to thirty millimetres of mercury, the loss during concentration is small. A small amount of the peroxide distils over with the water, the greater portion remaining in the distilling flask.

The commercial solutions have been found to contain some or all of the following impurities in small amounts: sulphuric, phosphoric, hydrochloric, and hydrofluosilicic acids, iron, aluminum, magnesium, barium, sugar, and glycerin. Of these impurities, the bases and hydrofluosilicic acid may be removed by a procedure the details of which would be out of place in this paper. The sugar, glycerin, and sulphuric or hydrochloric acid are, when present, added as preservatives. The phosphoric and hydrofluosilicic acids (and sometimes the sulphuric and hydrochloric acids)

[1] Recent experiments show that there is some reason for thinking that the germicidal power of peroxide solutions lies in the acid present, and not in the peroxide. This is important when it is considered that for other reasons a *neutral* solution is desirable.

are employed in the manufacture of the hydrogen peroxide from barium peroxide.

The acidity of the commercial solutions varies from 0.2 per cent. to 0.6 per cent. by weight, expressed in terms of sulphuric acid, and is a combination of all the acids present. In the purified and concentrated solution the amount of free acid may be much reduced. The greater the purity of the solutions the less acid is required to preserve them, and for a fifty-volume solution the amount required need not exceed 0.5 per cent., an amount insignificant in comparison with aromatic sulphuric acid, which is employed for purposes similar to that for which the peroxide is used.

The question naturally arises, if one cannot depend upon finding twenty-volume solutions in the trade which are of full value, what hope is there of finding fifty-volume solutions which are reliable? In this connection the following figures are presented, taken from a large number of instances which have come under my notice.

| Nature of Solution. | Acidity in terms of $H_2SO_4$. | Original Strength. | Time of Standing. | Tempera-ture. | Final Strength. | Per cent. loss. |
|---|---|---|---|---|---|---|
| | Per cent. | Vols. | Days. | | | |
| Commercial solution . . . . . . . . | 0.2 | 19.4 | 3 | 70°–80° F. | 19.4 | 0.0 |
| | | | 7 | 70°–80° F. | 19.2 | 1.0 |
| | | | 13 | 70°–80° F. | 19.2 | 1.0 |
| Commercial solution (repeatedly agitated) . . . . . . . . . . . . . | Approx. 0.2 | 23.0 | 60 | 70° F. | 20.0 | 13.0 |
| Commercial solution, concentrated at reduced pressure . . . . . . . . | 0.5 | 44.6 | 3 | 70°–80° F. | 44.6 | 0.0 |
| | | | 7 | 70°–80° F. | 43.5 | 2.4 |
| | | | 13 | 70°–80° F. | 43.0 | 3.6 |
| | | | 42 | 70°–80° F. | 40.4 | 9.4 |
| | | | 128 | 70°–80° F. | 27.9 | 37.4 |
| Commercial solution, concentrated at reduced pressure . . . . . . . . | 0.9 | 99.4 | 3 | 70°–80° F. | 100.0 | 0.0 |
| | | | 17 | | 82.7 | 16.8 |
| | | | 24 | | 76.0 | 23.4 |
| Commercial solution, concentrated at reduced pressure . . . . . . . . | 0.9 | 100.0 | 3 | 32° F. | 99.4 | 0.6 |
| | | | 16 | 32° F. | 98.6 | 1.4 |
| | | | 24 | 32° F. | 95.0 | 5.0 |

The figures show conclusively that it is possible to preserve even concentrated solutions for days or weeks without materially impairing their efficiency for dental purposes. It is obviously impossible for dealers to keep the concentrated solutions in stock for long periods, but if demand were made for solutions which were guaranteed to be freshly prepared, there is no satisfactory reason why the demand should not be supplied. It is also evident from the figures presented that the lower temperatures tend to increase the stability of the solutions, as does also protection from sunlight, dust, and mechanical agitation.

That the stronger solutions (fifty-volume or one-hundred-volume) may be employed without danger is shown by the fact that the strength has been increased to three hundred volumes in the treatment of certain cases of diphtheria without harmful effect upon the sound tissue.

All solutions, even the twenty-volume, produce white blotches if left for some time in contact with the skin, but these soon disappear. The more concentrated solutions frequently produce, also, a stinging sensation, lasting for few moments only, and causing no permanent discomfort.

The hydrogen peroxide is readily decomposed by contact with metallic surfaces, which renders its use in or with metallic instruments inadvisable.

In summarizing, it may be said that much of the lack of success attending the use of hydrogen peroxide has been due to the poor quality of the solutions obtainable; that the commercial solution, when of good quality, is serviceable as a cleansing agent, but that solutions of fifty-volume strength are much more efficient as detergents, and that such solutions may be preserved for a period of twenty to thirty days without losing their practical efficiency if moderate precautions are observed.

Since the preparation of this paper I have had occasion to examine two new brands hydrogen peroxide, the so-called pyrozone solution and that offered by the Oakland Chemical Company. The results may not be without interest, and, concerning the aqueous solutions, are briefly as follows: The acidity of these solutions has been reduced to about 0.06 per cent., which is practically neutral. On some grounds this is an advantage, but, as has already been intimated, it seems probable that the germicidal value is lessened.

The amount of impurity is very small.

It is an interesting fact that these solutions may be concentrated by careful evaporation in an open dish. In this way it is easy to obtain a fifty-volume solution, and it is possible to obtain two-hundred volume solutions with some sacrifice of material, but still in sufficient quantity to use in an emergency.

The hydrogen peroxide is easily soluble in ether, and the so-called five-per-cent. and twenty-five-per-cent. pyrozone solutions are ethereal. They are practically neutral.

If kept in ordinary glass-stoppered bottles, these solutions show an increase in strength, caused by the escape of the ether. This counterbalances any decomposition of the peroxide.

Experiments show that these ethereal solutions leave, on evaporation, a very concentrated solution of the peroxide in the small quantity of water which is always present. This residual solution is very caustic in its action even upon sound tissue.

There appears to be reason for supposing that the neutral aqueous solutions referred to above will retain their strength for a longer time than those mentioned in the table already quoted. The writer has as yet no positive data bearing upon this point.

